# Supplementary material for: Successful Bacteriophage-Antibiotic Combination Therapy against Multidrug-Resistant Pseudomonas aeruginosa Left Ventricular Assist Device Driveline Infection
Source: Viruses. 2023 May 20;15(5):1210. doi: 10.3390/v15051210 (PMC10223274; doi:10.3390/v15051210)
Supplement: Supplementary file 1 [file viruses-15-01210-s001.zip › viruses-2335741-supplementary.pdf]

Table S1. Phage therapy case reports for LVAD-associated infection treatment

N	Patient age and gender	Causative agent	Treatment Type	Phage(s) used	Treatment modality	Length of phage treatment	Adverse Events	Outcome, notes	City, country	Publication
1	65-year-old male	MSSA	Antibacterial and phage	AB-SA01 Cocktail consisting of 3 lytic *S. aureus* phages (Myoviruses)	I/v q12 (3 x 10^9 PFU/ml)	28 days	No adverse events	Clinical cure, bacterial persistence<br>* One week after phage therapy, the patient underwent successful heart transplantation; 7 months after transplantation, there was no recurrence of infection.	San Diego, California, USA	*Aslam,* 2019 [12]
2	67-year-old male	MSSA	Surgical, phage, antibacterial	Commercial phage product PYO phage cocktail consisting of Myoviruses Sb-1 and ISP active against *Staphylococcus* spp., *Streptococcus* spp., *Proteus* spp., *P. aeruginosa*, and *E. coli* and Staphylococcal phage Sb-1 (Myovirus) active against *Staphylococcus* spp. from ELIAVA	Topical application (1:1) of PYO) 10^6 PFU/ml, 5 ml) and Sb-1 (10^7 PFU/ml, 5ml) during surgery and through the drainage system in the surgical site q8	10 days	Mild nausea.	Clinical cure, bacterial eradication.	Berlin, Germany	*Mulzer,* 2019 [8]
3	61-year-old male	*S. aureus*	Antibacterial and phage	Myovirus CH1 against *S. aureus*	Topical application through a chest tube inserted during surgery (1 x 10^9 PFU/ml, 20 ml) q12	7 days	No adverse events.	Clinical cure, bacterial eradication.<br>*20 months later, the patient died from heart transplant failure.	Hannover, Germany	Rubalskii, 2020 [13]
4	51-year-old male	*S. aureus*	Antibacterial and phage	Four phages against *S. aureus* – Myoviruses CH1, Sa30, SCH1, and SC111	Intra nasal 2 ml q24, oral 10 – 20 ml q24, topical through drainage 10 ml q24 (8 days), 10 – 20 ml q12 (6 days) (1 x 10^9 PFU/ml	14 days	No adverse events.	Treatment failure, the patient died 1.5 months later of *S. aureus* sepsis.<br>**S. aureus* eradication was observed from the nose and throat, decrease in bacterial load in drainage fluid	Hannover, Germany	Rubalskii, 2020 [13]
5	60-year-old male	MDR *P. aeruginosa*	Antibacterial and phage	GD-1, consisting of 3 phages	I/v q8 (1.9 x 10^7 PFU/ml)	6 weeks	No adverse events.	Treatment failure, development of bacteremia in the second	San Diego, California, USA	Aslam, 2020 [9]

								week of treatment, and discharge from the driveline after treatment.<br>*Phage-specific neutralising serum developed during bacteremia in the second treatment week.		
6	82-year-old male	*P. aeurginosa*	Surgical, phage, antibacterial	Episode 1: SDSU1 (2 phages PAK-P and E217) SDSU2(2 phages PAK_P1, PAK_P5) cocktails.<br><br>Episode 2: PPM3 (4 phages) cocktail.	Episode 1: I/v q8 (2 x 10^5 PFU/ml) q12 (7.58 x 10^5 and 4 x 10^{10} PFU/ml). Single topical application during surgery<br><br>Episode 2: I/v q12 (1 x 10^9 PFU/ml)	Episode 1: 73 days<br><br>Episode 2: 4 weeks	Fever, wheezing, and shortness of breath using SDSU1 phage at a concentration of 1 x 10^{11}PFU/ml. The phage was well tolerated when the concentration was reduced to 10^{10} PFU/ml.	Treatment failure developed recurrent bacteremia during episode 1 in the last week of treatment. During episode 2, bacteremia was present in the fourth week of treatment.	San Diego, California, USA	Aslam, 2020 [9]
7	53-year-old male	MDR *P. aeruginosa*	Surgical, antibacterial, and phage	Three lytic PA phages (Podovirus PNM; Myoviruses PT07 and 14/1)	I/v 7h infusion (10^7 PFU/ml, 10 ml/h)<br><br>Topical q12h (10^8 PFU/ml, 50 ml) by dressing and directly on the wound during surgery	5 days	Increase in gamma-glutamyl transferase (GGT) and direct bilirubin. After the reduction in phage dosage (0.5 log PFU/ml from initial titer), liver damage markers did not increase.	Clinical cure, bacterial eradication.<br>*4 months later, the patient died from a noninfectious cause.	Berlin, Germany	*Tkhilaishvili*, 2021 [10]
8	68-year-old male	MDR *P. aeruginosa*	Antibacterial and phage	Autophage and Pyo phage cocktail from ELIAVA	Topical (dressing) application of Autophage (10^8 PFU/ml) and Pyo (10^6 PFU/ml) q12	12 days Autophage and 5 days Pyo	No information available.	Recurrence of *P. aeruginosa* infection with development of phage resistance.	Berlin, Germany	*Tkhilaishvili*, 2022 [14]
9	57-year-old male	MSSA and *Proteus mirabilis*	Surgical, phage	SniPha 360 commercial cocktail of lytic phages against *Escherichia coli, Staphylococcus aureus, Pseudomonas aeruginosa, Streptococcus pyogenes, Proteus vulgaris* and *Proteus mirabilis*	Single topical application during surgery of viscous phage-containing fluid (20 mL SniPha 360 (1 × 10^7 PFU/ml) mixed	1 day (single application)	No information available.	Recurrence of MSSA infection 2 months later, eradication of *P. mirabilis.*<br>* *in vitro* testing of the phage cocktail showed lytic activity only against	Rostock, Germany	Püschel, 2022 [15]

					with saline and polysaccharide			*P. mirabilis*; no lytic effect was observed on *S. aureus*		
10	51-year-old male	MSSA	Surgical, phage, antibacterial	SniPha 360 commercial cocktail	Single topical application during surgery of viscous phage-containing hydrophilic galenic (20 mL SniPha 360 (1 × 10^7 PFU/ml)	1 day (single application)	No information available.	Recurrence of MSSA infection 6 months later.	Bochum, Germany	Rojas, 2022 [11]

MDR – multidrug-resistant; MSSA – methicillin-sensitive *Staphylococcus aureus*; AB-SA01 - AmpliPhi Biosciences Corporation

Biofilm formation of used bacterial strains.

The isolated bacterial strain PAP01 was a weak biofilm producer; however, the reference strain was a strong biofilm producer already after 2 h of incubation. There were significant differences between both strains at all incubation times, $p < 0.0001$, with regard to the formation of the biofilm (Figure S2).

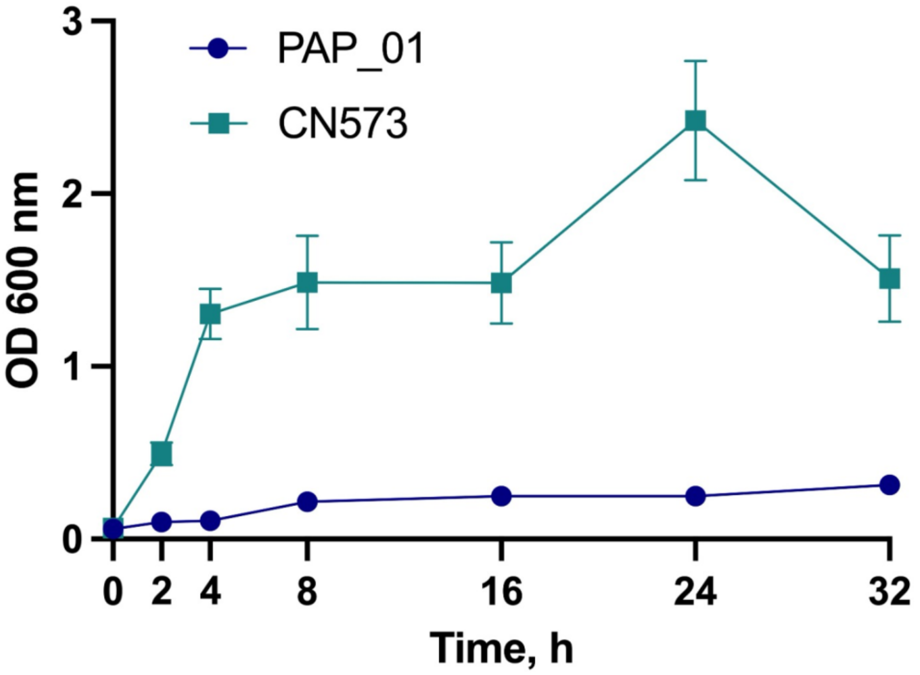

Figure S1. Biofilm formation of P. aeruginosa PAP01 and CN573 strains using crystal violet assay.

Phage effect against planktonic cells, biofilm eradication, prevention of biofilm formation, and bacterial resistance to phages in CN573

In the biofilm eradication model by strain CN573, the effect of phages PNM and PT07 and both combined showed a significant absence of growth in planktonic cells at all concentrations tested after 12 and 24 h, and showed a significant eradication of biofilms (Figure S3). The results show that in some cases, the suppression of bacterial planktonic cell growth after 12 h was less when higher concentrations of phages were applied. However, this was not observed in planktonic cells after 24 h, nor for biofilm eradication. When applying both phages together, there were no concentration dependent differences in the bacterial growth curves and MBEC values. The selection of phage resistance was observed only in MBEC plate, and only against PNM phage at 10^7 PFU/ml (Figure S3 C).

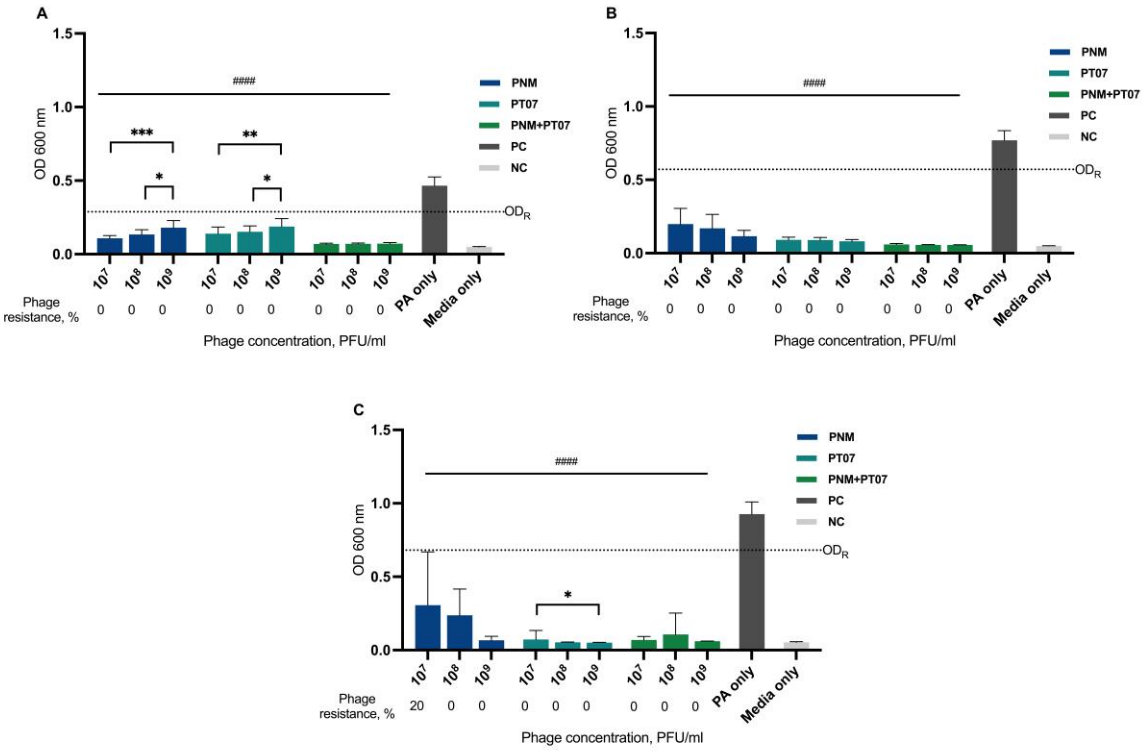

Figure S2. Bacterial growth suppression and MBEC of PNM, PT07, or both combined, against CN573 in a biofilm eradication model. The bars represent mean values + standard deviations. The number of phage-resistant strains in % is represented below the bars. (A) Bacterial growth suppression after 12 h; (B) Bacterial growth suppression after 24 h; (C) MBEC; PC – positive control, untreated CN573; NC – negative control, media only; ODR line represents the cut-off value to determine phage resistance; Bars with a hash sign are statistically different from the positive control, and bars with an asterisk represent the statistical difference between concentrations of the same phage; */# p-value < 0.05, **/## p-value < 0.01, ***/### p-value < 0.001 and ****/#### p-value < 0.0001.
